# Milk miRNA expression in buffaloes as a potential biomarker for mastitis

**DOI:** 10.1186/s12917-024-04002-1

**Published:** 2024-04-20

**Authors:** Abhishek B. Jadhav, Shailesh D. Ingole, Simin V. Bharucha, Korsapati L. Yoshitha, Rajiv V. Gaikwad, Rajesh R. Pharande, Shambhudeo D. Kharde

**Affiliations:** 1 Department of Veterinary Physiology, Mumbai Veterinary College, Maharashtra Animal and Fishery Sciences University, Mumbai, India; 2 Teaching Veterinary Clinical Complex, Mumbai Veterinary College, Maharashtra Animal and Fishery Sciences University, Mumbia, India; 3 Department of Veterinary Microbiology, Mumbai Veterinary College, Maharashtra Animal and Fishery Sciences University, Mumbai, India

**Keywords:** Milk, Mastitis, miRNA, Buffaloes

## Abstract

**Background:**

Buffaloes have the highest potential for production due to a promising gene pool that is being enhanced and upgraded. Mastitis is a significant health impediment that greatly diminishes milk yield and quality, affecting rural farmers’ livelihoods. The traditional gold standard used for diagnosing mastitis or subclinical mastitis is CMT, but it has the drawback of false positive or negative results. Subclinical mastitis, if not treated promptly, can lead to mammary tumors. To address the gap in early diagnosis of subclinical mastitis in CMT-negative milk of buffaloes, we performed a retrospective analysis and evaluated the milk miRNA expression profiles as potential biomarkers.

**Results:**

Thirty buffalo milk samples based on clinical signs and CMT were divided into normal, subclinical, and clinical mastitis. SCC evaluation showed significant differences between the groups. The data analysis demonstrated that the elevation of miR-146a and miR-383 differed substantially between normal, subclinical, and clinical mastitis milk of buffaloes with 100% sensitivity and specificity. The relationship of SCC with miR-146a and miR-383 in normal/healthy and subclinical mastitis was positively correlated.

**Conclusion:**

The overexpression of miR-146a and miR-383 is associated with inflammation. It can be a valuable prognostic and most sensitive biomarker for early mastitis detection in buffaloes with SCC below 2 lakhs and CMT-ve, enhancing the accuracy of subclinical mastitis diagnosis.

**Supplementary Information:**

The online version contains supplementary material available at 10.1186/s12917-024-04002-1.

## Introduction

India has the most buffaloes in the world, and milk production is the backbone of the Indian dairy industry. Nevertheless, much more can be done to improve India’s buffaloes. Compared to cows, buffaloes contribute more than half of the total milk production in the country [1]. Mastitis is a significant health issue for dairy animals, with major impacts on dairy production, resulting in substantial economic losses for the dairy industry [2].

Bovine mastitis is an inflammatory disease divided into subclinical and clinical types. Unfortunately, the most common type of mastitis is subclinical, which explains why it is quickly disseminated to other animals in the herd during regular milking [3]. Somatic cell count and bacteriological culturing are frequently used to diagnose bovine mastitis [4]. The most commonly used cow side test is the California Mastitis Test (CMT), which estimates somatic cell count. Subclinical mastitis often appears asymptomatic but persists throughout the entire lactation period or the animal’s life [5]. Clinical mastitis can result in 10% or more milk loss on a quarter basis and a 10% reduction in the remaining lactation. In contrast, subclinical mastitis causes two-thirds of milk production losses [6]. Subclinical mastitis has been reported to be more important (5–20% in buffaloes) than clinical mastitis because it is 15–40 times more prevalent than clinical mastitis [7]. Subclinical mastitis requires special diagnostic tests for detection at early stages. Other factors that influence milk somatic cell count, besides infection, such as parity, season, and stress, can affect CMT limitations and how the results are interpreted by different testers [8].

The inflammatory response in mammals is a complex and rapid physiological response to noxious stimuli such as microbial pathogens. Recent evidence also points to a critical role for a specific class of noncoding RNAs, called microRNAs (miRNAs), in managing certain inflammatory process features and significantly impacting their magnitude [9]. Many diseases, including inflammation, have been linked to miRNAs. miRNAs have been identified in cells and fluids such as saliva, amniotic fluid, blood, urine, and milk [10]. It has been claimed that 95% of the miRNAs expressed in human milk are also described in bovine and goat milk and that bovine milk pasteurization does not seem to destroy miRNA [11]. MicroRNAs in milk have been used as a novel method of quality control and disease biomarker [12]. Exosomes and milk fat globules transfer milk miRNAs from mammary gland epithelial cells [13].

miR-146a and miR-383 have more gene targets related to inflammatory pathways than other miRNA candidates [14]. miR-146a is well characterized, and the first reported inflammation-related miRNA [15]. Human miR-146a regulates the innate immune response by targeting TNF receptor-associated factor 6 (TRAF6) and interleukin-1 receptor-associated kinase 1 (IRAK1), and bovine miR-146a is homologous to human equivalents [16]. miR-146a is a regulator in the immune mechanism and inhibits the function of regulatory T cells [17]. However, its functional role and expression pattern in subclinical and clinical mastitis in buffaloes has not been reported. miR-383 has been reported to correlate with various inflammatory diseases. It may participate in regulating the immune response similar to miR-146a and may have an essential function in controlling inflammatory diseases. miR-383 has been reported to be dysregulated in various human cancers, and its expression is markedly changed during tumorigenesis. It also has an oncogenic function in promoting proliferation, enhancing metastasis, and inducing tumorigenesis [18].

The differences in miRNA expression levels between healthy animals and patients are a significant study area for disease-related miRNAs. CMT provides an inexpensive, quick, and easy procedure, but its subjectivity makes it less accurate and reliable when used alone. CMT can be performed for SCC estimation but has the drawback of false positive/negative results [19]. The application of miRNAs as biomarker entities has shed light as a tool for detection and verification at the disease level in animals but has yet to be widely studied. The differences in milk miRNA expression levels may depend on subclinical, clinical, or healthy buffaloes, which is largely unknown. The indicators of bovine mastitis may have utilized the presence of microRNAs in milk as a potential biomarker [20], and comparatively, milk miRNA expression is 2-fold higher than serum miRNA [12]. Several miRNAs from bovines have been reported, and their expression profiles may be used as potential biomarkers for the diagnosis, prognosis, and therapy of mastitis. miRNA expression in biofluids can provide stable and disease-specific biomarkers [21]. Exosomal miRNAs in buffalo milk have been thoroughly analyzed, uncovering their wide-ranging spectrum [22]. The miRNAs in milk are found to impact disease resistance, immune response, and fundamental metabolism in water buffaloes [23]. Thus, research on mastitis in bovines needs to be extended to the panel of inflammatory or mammary tumor markers by adding new markers, which helps in diagnostic and therapeutic purposes. Therefore, this study aimed to evaluate the expression of miRNA 146 and miRNA 383 as a specific and sensitive biomarker for mastitis and pinpoint the diagnosis and prognosis of subclinical mastitis in buffaloes at an early stage compared to CMT.

## Materials and methods

### Milk collection, processing, and storage

The buffalo milk samples were collected from privately owned farms in the Mumbai region (Aarey Milk Colony, Goregaon, Mumbai, India) that tested positive for mastitis, including CMT + ve (Subclinical Mastitis) and CMT –ve (Normal). A total of 30 Murrah buffalo milk samples were included in the study. The animals were selected irrespective of lactation yield, stage, and parity and based on symptoms, physical examination of the udder, and CMT. Accordingly, the buffalo milk was divided into three groups (10 each) viz. Clinical Mastitis, Subclinical Mastitis, and CMT –ve (Normal). Milk samples (30–50 ml) were collected aseptically at the milk collection point, where the milker milked the buffalo and transferred the milk to provided tubes (free of RNase and DNase), which were promptly tested with the DeLaval CMT Kit. The sample was judged negative for mastitis if no gel formation was seen in any quarter. The sample was considered positive for mastitis if gel formation was observed in any quarter. After CMT, the milk samples were kept at 4 °C until transport to the laboratory, where they were centrifuged at 3000×g for 15 minutes to remove fat, casein, and cell debris and then spun again at 15000×g for 15 minutes to recover the whey/aliquot, which was then stored at − 80 °C for RNA extraction.

### miRNA (miR-146a and miR-383) expression

#### Isolation of miRNA

Isolation of miRNA from 300 μl whey/aliquot was performed using the mirVana™ PARIS™ RNA and Native Protein Purification Kit (Thermo Fischer Scientific, Invitrogen, Vinius, Lithuania) and the instructions provided by the manufacturer. The concentration and purity of the miRNA solution were determined by measuring its absorbance at 260 and 280 nm (Additional files 1 and 2).

#### Reverse transcription of miRNA

The miRNA was reverse transcribed to cDNA using the PrimeScript 1st strand cDNA synthesis kit (Takara Bio Inc., Shiga, Japan, Cat. # 6110A) and the instructions provided by the manufacturer. For reverse transcription, we used TaqMan Small RNA Assays**.** The assay uses a stem-looped primer for reverse transcription to accurately detect mature miRNAs.

#### qRT-PCR

Quantitative expression of miR-146a and miR-383 were studied using a TaqMan real-time PCR (qRT-PCR) assay. qRT-PCR was performed using TaqMan Fast Advanced Master Mix (Fischer Scientific, Applied Biosystems, Vilnius, Lithuania, Cat no: 4444556) and a CFX 96™ PCR system (Bio-Rad Laboratories, Inc., Hercules, CA, USA). miR-92a was used as a reference gene [24] for the miRNA expression studies and comparative experiments between normal, subclinical, and clinical mastitis milk of buffaloes. The TaqMan miRNA-specific primers and probe sequences for miR-146a and miR-383 published (Lai et al., 2017b) were used, and their IDs were miR-383 (ID: 000573), miR-92a (ID: 000431), and miR-146a (ID: 005896_mat). The TaqMan microRNA assay and cDNA templates were thawed on ice, vortexed gently, and then centrifuged briefly to bring the contents to the bottom of the tube. A 20 μl PCR mixture was prepared (Table 1).

**Table 1:** PCR reaction mix

Component	96-well plate (0.1 ml standard)	Final Concentration
Taqman® Fast Advanced Master Mix (2x)	10.0	1x
Taqman® Assay Primer/Probe (20x)	1.0	1x
cDNA template	2.0	0.001–100 ng/well
Nuclease free water	7.0	–
Total Volume per reaction	10.0	20.0

#### miR-146a, miR-383 and miR-92a

Thermal cycling conditions for miR-146a, miR-383, and miR-92a were 95 °C for 20 seconds (polymerase activation) followed by denaturation at 95 °C for 3 seconds and ending with annealing/extension step at 60 °C for 30 seconds for 40 cycles. After thermal cycling, the Cq or Ct value was noted.

#### Expression assay

The gene expression levels of miR-146a, miR-383, and miR-92a in the milk of buffaloes with clinical mastitis, subclinical mastitis, and normal/healthy buffaloes were compared. The average C_T_ value was calculated for each sample by taking the mean of the C_T_ values obtained from technical duplicates. Using the 2^-ΔΔC^_T_ method [25], the relative difference in the expression level of a target miRNA was determined.

#### Statistical analysis

Data analysis was performed using IBM SPSS version 22. The data were compared using one-way ANOVA. AUC-ROC curve analysis was used to calculate the sensitivity and specificity of miR-146a and miR-383. The correlation coefficients were calculated to study the relationship between SCC, miR-146a, and miR-383.

## Results

The milk was collected according to the clinical observation of buffaloes. Those that showed blood in milk and pus in milk with a foul smell were considered the clinical mastitis group, whereas buffaloes that were screened with CMT and showed positive results were grouped as a subclinical mastitis group, and buffaloes that were screened and showed no reaction to CMT were grouped as the normal animal group. The sample size was 30 milk samples, with 10 in each group.

### Somatic cell count

The mean SCC in normal, subclinical, and clinical mastitis milk was 0.93 ± 0.08 × 10^5^ cells/ml, 10.64 ± 1.82 × 10^5^ cells/ml, and 37.25 ± 2.8 × 10^5^ cells/ml, respectively**.** The SCC ranges in normal, subclinical, and clinical mastitis milk of buffaloes are 0.56 to 1.45 (× 10^5^ cells/ml), 4.25 to 19.06 (× 10^5^ cells/ml) and 24.03 to 48.73 (× 10^5^ cells/ml), respectively (Additional file 3). Data analysis revealed that the mean SCC in normal, subclinical, and clinical mastitis milk differed significantly **(***P* < 0.000).

### miR-146a expression

**Fig. 1:**
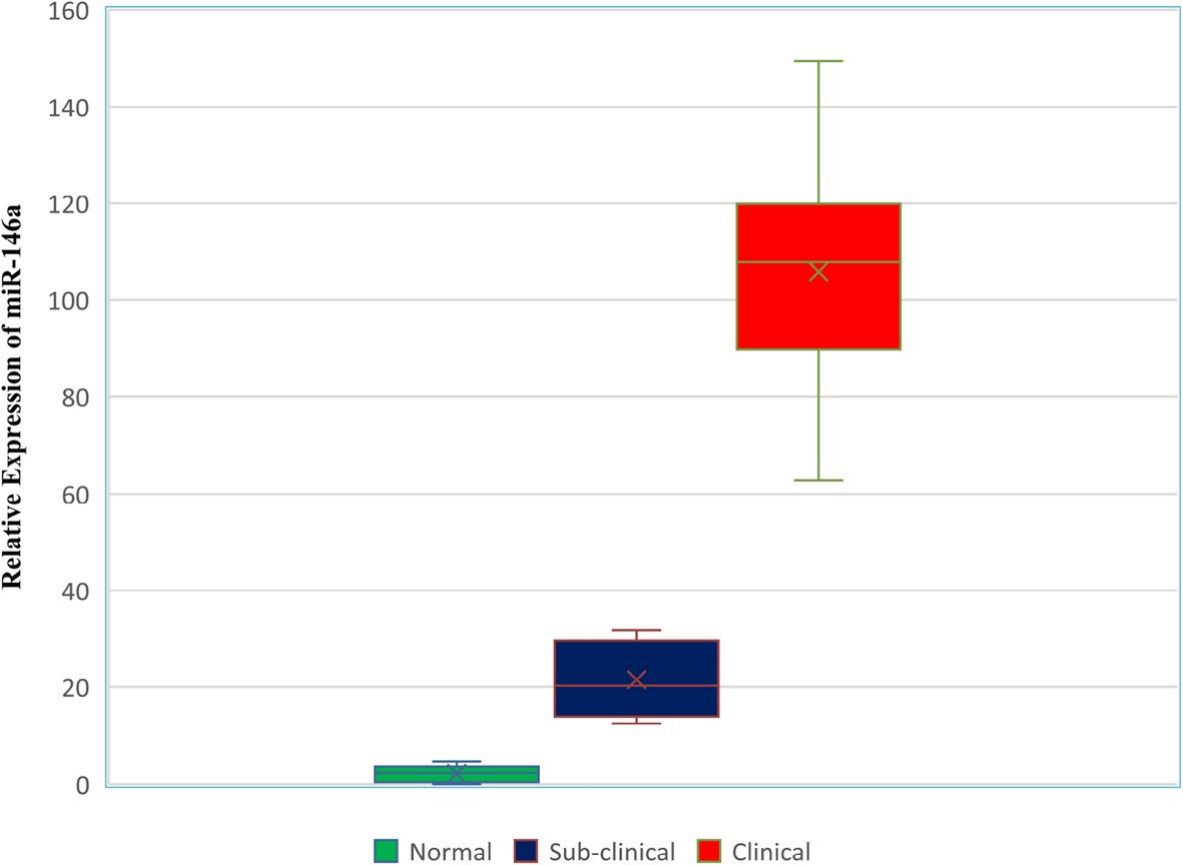
Box and Whisker plots for miR-146a expression in milk by qRT-PCR between normal, subclinical, and clinical mastitis buffaloes. Horizontal lines in the box are median values, and X are mean values

Relative expression fold changes for milk samples of buffaloes with clinical mastitis, and subclinical mastitis were calculated using the formula (2^-ΔΔCt^). miR-146a was upregulated in all the test samples. The average expression fold changes in normal/healthy, subclinical mastitis, and clinical mastitis milk of buffaloes were 2.45, 21.52, and 105.86 fold, respectively (Fig. 1). Expression fold change values ranged from 12.41 to 31.85 in buffaloes with subclinical mastitis and 62.81 to 149.40 in buffaloes with clinical mastitis. miR-146a expression was increased in the milk of buffaloes with subclinical and clinical mastitis. Comparatively, clinical mastitis showed a more significant expression level than subclinical mastitis. The data analysis demonstrated that the elevation of miR-146a differed substantially (*p* < 0.000) between control/healthy, subclinical, and clinical mastitis milk of buffaloes (Additional files 4, 5, 6, and 7).

### miR-383 expression

**Fig. 2:**
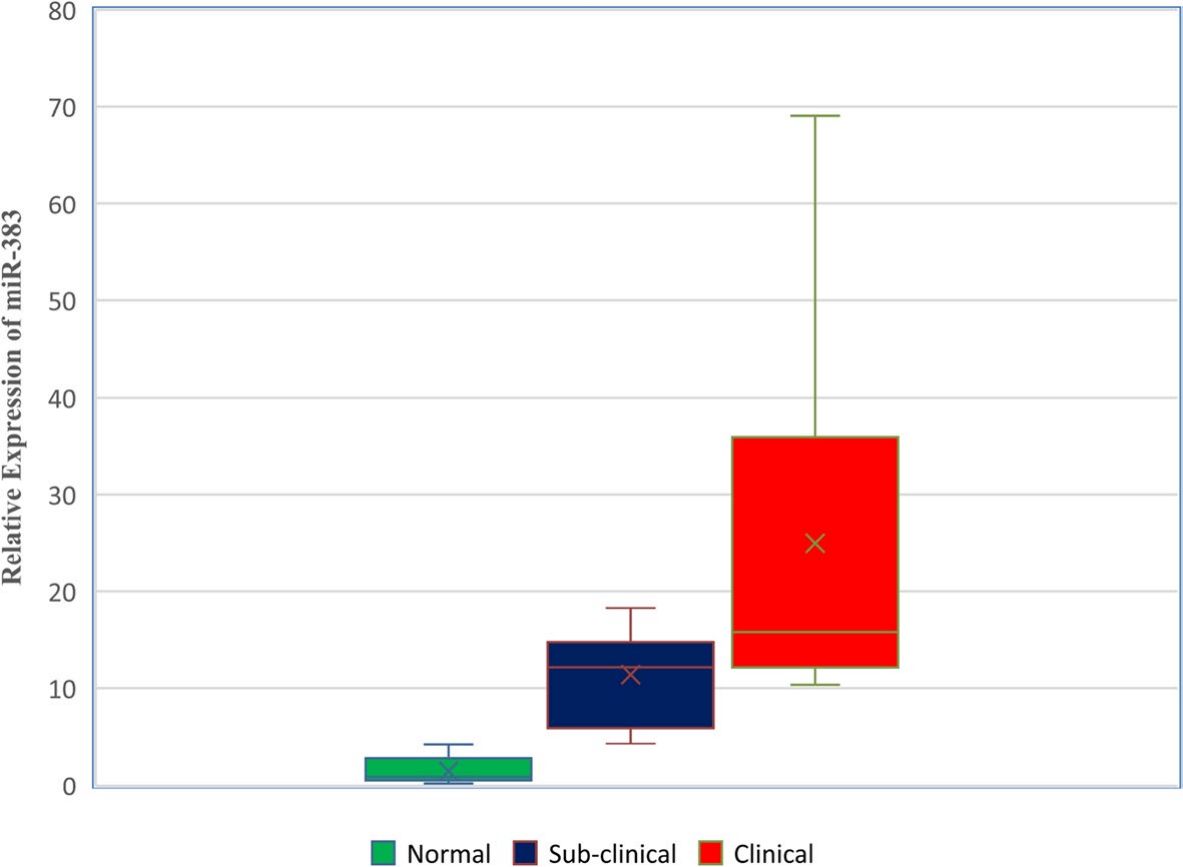
Box and Whisker plots for miR-383 expression in milk by qRT-PCR between normal, subclinical, and clinical mastitis buffaloes. Horizontal lines in the box are median values, and X are mean values

miR-383 was upregulated in all the test samples. The average miR-383 expression fold changes in normal milk, with subclinical mastitis, and with clinical mastitis milk of buffaloes were 1.48, 11.37, and 24.97 fold, respectively (Fig. 2). Expression fold change values ranged from 4.32 to 18.25 in buffaloes with subclinical mastitis and 10.34 to 69.07 in buffaloes with clinical mastitis. miR-383 expression was increased in the milk of buffaloes with mastitis. Clinical mastitis had a greater expression level than subclinical mastitis. The data analysis demonstrated that the elevation of miR-383 differed substantially (*p* < 0.05) between buffaloes with subclinical and clinical mastitis and control/healthy buffaloes’ milk (Additional files 8, 9, 10 and 11).

### ROC analysis for miR-146a and miR-383

**Fig. 3:**
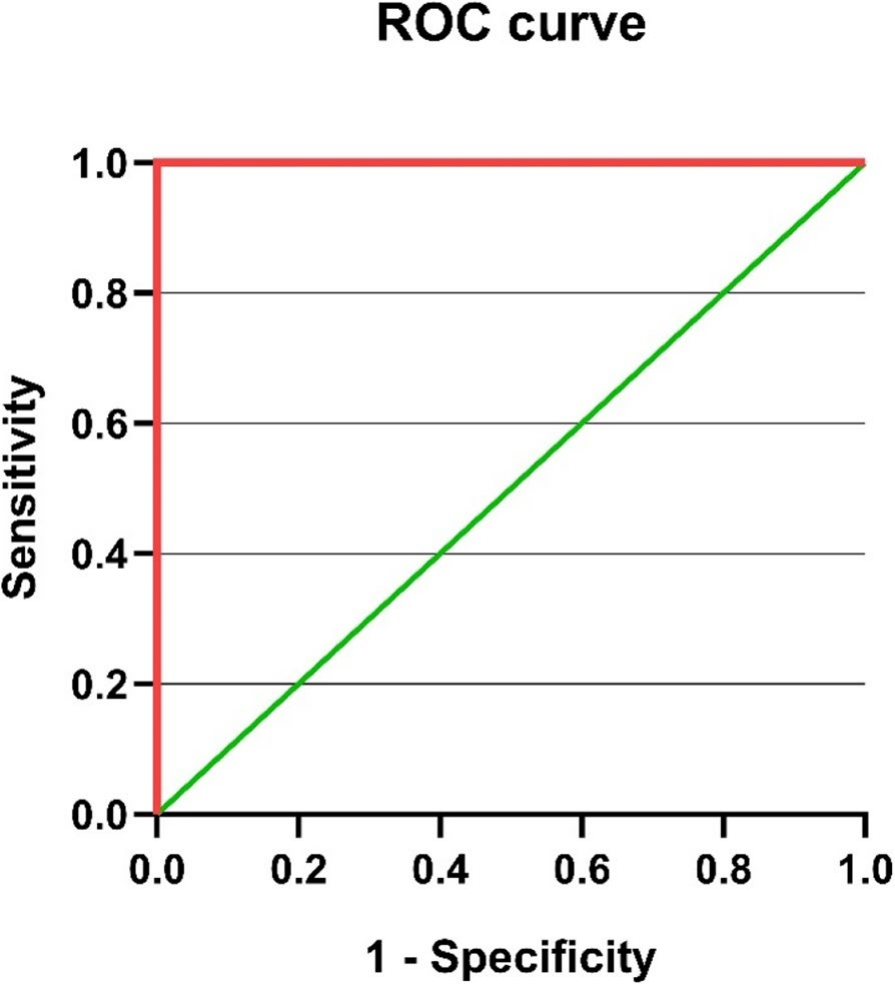
ROC curve for miR-146a in the clinical mastitis group

**Fig. 4:**
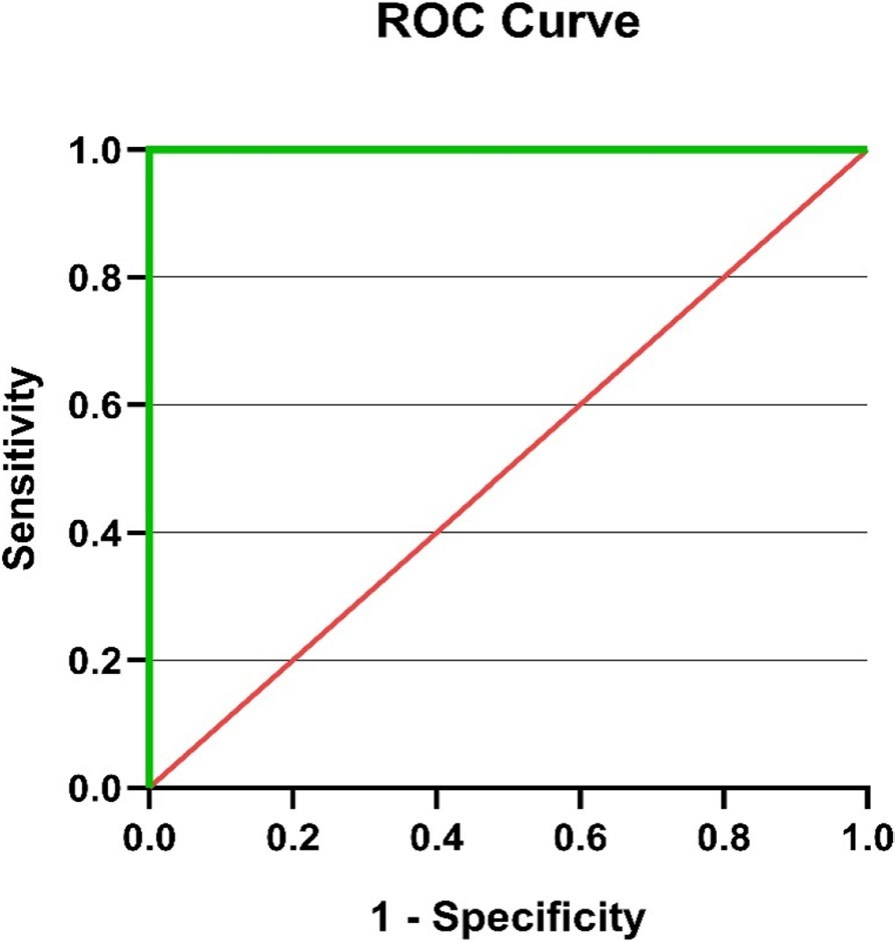
ROC curve of miR-146a in the subclinical mastitis group

**Fig. 5:**
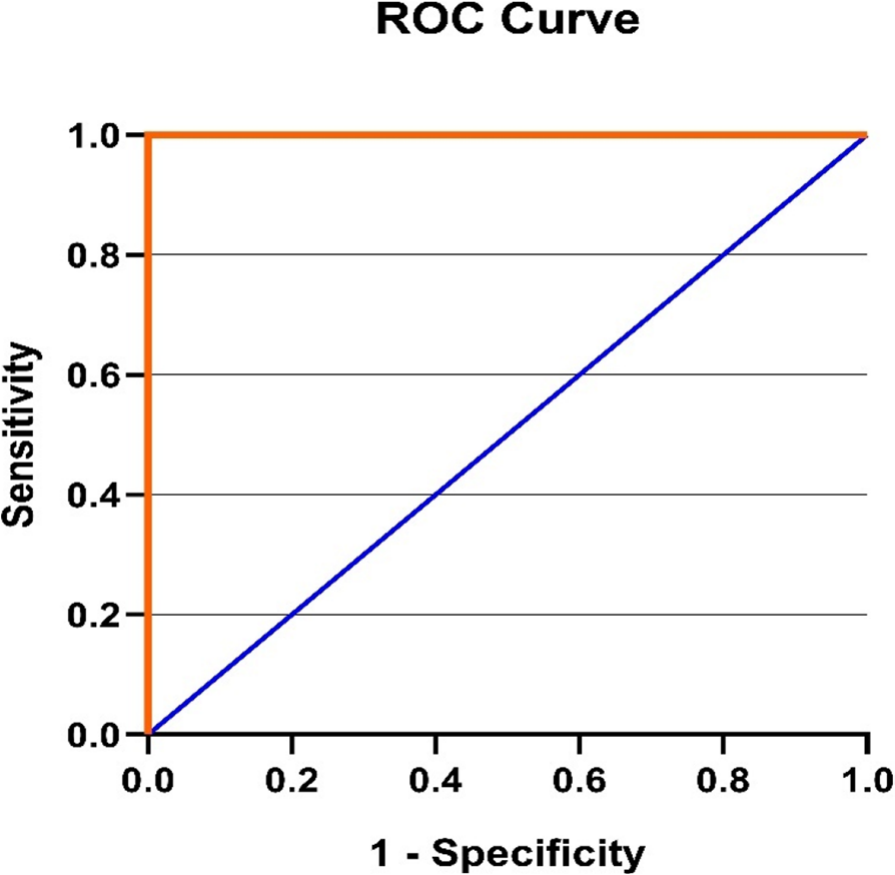
ROC curve of miR-383 in the clinical mastitis group

**Fig. 6:**
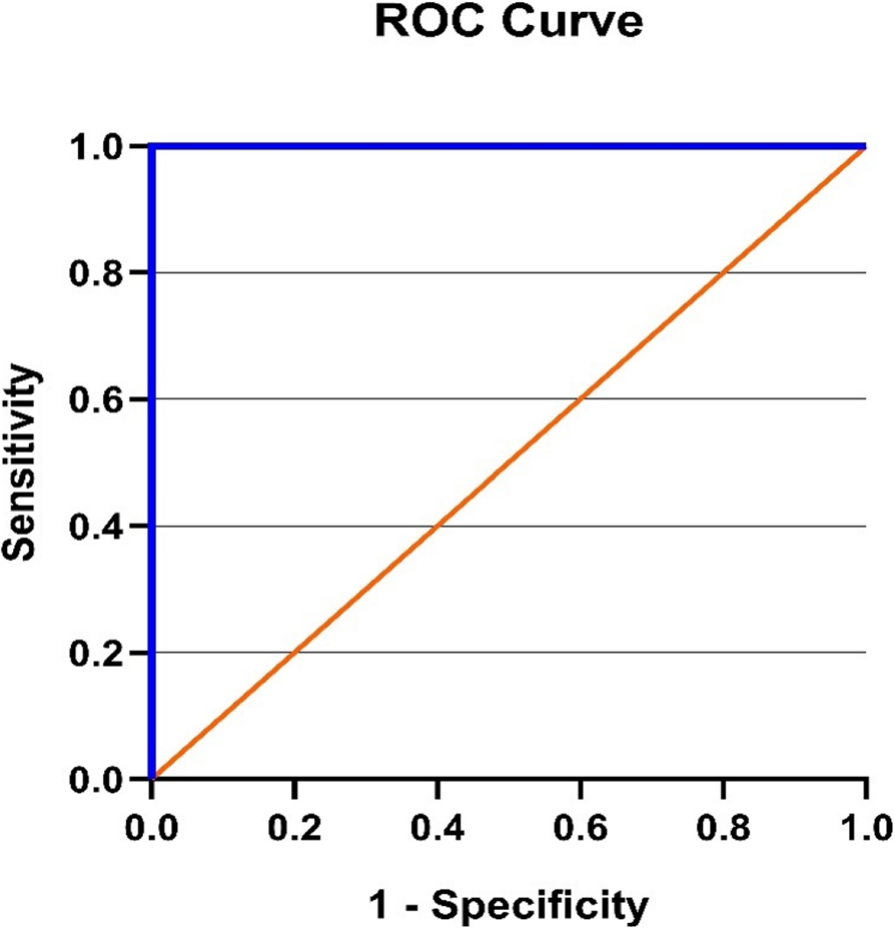
ROC curve of miR-383 in the subclinical mastitis group

The potential of the miRNA to differentiate between the normal, subclinical, and clinical groups was assessed using a receiver operating characteristic curve analysis of relative expression levels of miR-146a and miR-383 to explore further the diagnostic accuracy of miRNA (Figs. 3, 4, 5, 6). The best cut-off point, sensitivity, and specificity of each miRNA were determined using the area under the curve analysis and the Youden index. miR-146a and miR-383 had high predictive values (AUC = 1) with specificity and sensitivity of 100%, respectively.

### Correlation of somatic cell count with miR-146a and miR-383 expression

The relationship between SCC and miR-146a and miR-383 expression in the milk of normal buffalo milk was positively correlated. Similarly, a positive correlation was observed between SCC and mR-146a and miR-383 expression in subclinical mastitis, whereas a negative correlation was observed in the clinical mastitis milk of buffaloes. This is the first study where SCC is correlated with miRNAs (Additional files 12, 13 and 14). Milk somatic cell count is a sensitive indicator of mammary gland inflammation.

By comparing SCC with milk miRNA expression, it was observed that the miR-146a and miR-383 fold changes were between 2 and 4 fold in buffaloes with SCCs from 0.56 × 10^5^ to 1.45 × 10^5^ cells/ml. The SCC in subclinical and clinical mastitis was above 4 lakhs, and the fold change in miR-146a expression was between 21.52 and 105.86, and for miR-383, it was 11.5 and 24.97 fold, respectively.

## Discussion

The potential of miR-146a and miR-383 expression as biomarkers and its correlation with SCC to diagnose subclinical mastitis in very early stages were explored in the current experiment. The significant increase in SCC in normal, subclinical, and clinical mastitis milk increases the probability of inflammation or infection. The result of the SCC in the present study is in agreement or slightly higher with previous studies [26–31] in cows and buffaloes, which may be due to environmental stress, season and quarter capacity of buffaloes, variation between animal age, their lactation period and parity. These reasons can contribute to the fluctuations in somatic cell counts in normal cows and buffaloes. According to Sri Balaji et al. (2016) [31], threshold variation also plays an important role.

The SCC in subclinical and clinical mastitis in the present study was higher than those reported [28], which may be due to the small sample size, causative agent, and environmental conditions. The low somatic cells/ml of milk from buffaloes with clinical mastitis [27] is relatively low compared to our findings, as the first stage of lactation, last dry period, parity, and bedding material are risk factors that lead to mastitis. The lower SCC in subclinical mastitis than in clinical mastitis in riverine buffaloes can be because the average number of composite milk SCC grows as the number of contaminated quarters increases, and there is a significant input of PMN into the milk [32].

The mean SCC in subclinical and clinical mastitis was significantly higher (*P* < 0.05) than normal. The average range was very high compared to the count reported [30, 31] in buffaloes and dairy cows, and there is a positive correlation between milk yield and clinical mastitis [33, 34]. The affected quarters are in chronic inflammation, eventually leading to a higher somatic cell count, which could adversely affect the milk-producing tissues [35].

### miR146a

miR-146a expression was increased in the milk of buffaloes with subclinical and clinical mastitis. Comparatively, clinical mastitis showed a greater expression level than subclinical mastitis. The data analysis demonstrated that the elevation of miR-146a differed substantially (*p* < 0.000) between buffaloes with subclinical and clinical mastitis and control/healthy buffaloes’ milk. Similar findings were observed with significant upregulation in miR-146a in CMT + ve milk of cows, which is known to be related to inflammation [8, 21] and provides accurate inflammatory indicators in mammary glands. This suggests that the inflammation-related miRNA miR-146a in buffalo milk is affected by mastitis. The study [36] showed altered miRNA expression in mammary epithelial cells of mastitis-affected cows. Our study showed similar alterations in miRNA expression but in buffaloes’ subclinical and clinical mastitis milk. The present study correlates with Lai et al. (2017b) [8], and milk is a suitable liquid biopsy prognostic biomarker similar to blood and urine, which can be used as a non-invasive, safe, and fast method for predicting mastitis. The results show that miR-146a can potentially be a liquid biopsy biomarker for bovine mastitis. miR-146a expression is upregulated in mammary tissues of Holstein cows with subclinical and clinical mastitis by two to three folds, respectively [16]. They suggested that miR-146a plays a major role in the immune response of mammary inflammation and suspected that it regulates target genes such as TRAF6 and IRAK1. Moreover, miR-146a expression in the milk of mastitis-affected cows was studied [37], and they observed upregulation of miR-146a, which was the case for our research. Similar to our results, Ozdemir (2020) [14] also found that the relative expression of miR-146a was upregulated in mastitis milk samples compared to normal milk samples (*P* < 0.05). miR-146a is a well-known inflammation-related miRNA, and increased miR-146a is essential for innate immunological or inflammatory brain cell responses in prion-mediated infections [38].

This has been implicated in the Toll-like receptor 4 and Nuclear Factor Kappa B (TLR4-NFkB) signaling pathway immune response. miR-146a works to demote the immune response (Faisal and Naoki 2020) [37]. The LPS-mediated elevation of miR-146a occurs in an NF-kB-dependent manner. Identifying IRAK1 and TRAF6 as target genes of miR-146a post-translational repression suggests a novel mechanism of negative feedback regulation of TLR and cytokine receptor signalling. The TLRs that recognize bacterial constituents and reside on the cell surface trigger miR-146a induction [15]. Thus, mastitis is a bacterial infection, and we observed unregulated expression of miR-146a. The aberrant expression of miR-146a might be linked to the development of cancerous phenotypes [39]. Most of the studies attenuating the mechanism of inflammation involve negative transcriptional loops. However, miRNAs are an addition to the negative regulation of inflammation, and they are a feedback system in which a bacterial component, as in mastitis, induces NF-kB through an MYd88-dependent pathway, resulting in miR-146a upregulation [15].

miR-146a is highly upregulated in tolerized cells and acts as a tuning mechanism to present an overstimulated inflammatory state, and modulating the level of miR-146a can be used in therapeutic interventions for inflammation [40]. Similarly, it will help protect against mastitis in the early/initial stages to suppress breast cancer metastasis [41]. Moreover, evidence suggests that inflammation and cancer are intimately linked [42]. miR-146a may inhibit lipid uptake by blocking the TLR4-SRC-FAK-JNK axis and inhibiting the phosphorylation of Pyk2 and paxillin [43]. Thus, due to inhibitory lipid uptake, there is an increase in the levels of lipids in the omega 6 and omega 3 lines, which can be one of the reasons for the flocculation of milk in mastitis.

miR-146a downregulates TRAF6 and NF-kB expression post-transcriptionally by directly targeting the 3’UTR of TRAF6, consequently suppressing the production of the inflammatory mediators TNF alpha, IL-6, and IL-8. This suggests that miR-146a acts as a negative feedback regulator of inflammation by downregulating the TLR4/TRAF6/NF-kB pathway. This indicates a regulatory mechanism of miR-146a on the immune response of bovine mammary infection and may also provide a potential therapeutic target for mastitis [44].

In contrast, Srikok et al. (2020) [45] observed that the relative expression of miR-146a in healthy animals was significantly higher than in milk from subclinical and clinical mastitis animals. This disparity might be attributed to using different housekeeping miRNA genes (miR-92a vs miR-16b) in the research and the temporal dynamics of the progress of the infection.

Milk miR-146a as a biomarker has led to interesting findings in the context of infection. This is a good target for monitoring infection status, as it is stable in milk, and its levels are impacted by mammary gland status. These changes in miR-146a observed in normal, subclinical, and clinical mastitis milk are essential in health and disease, especially during infection and cancer. Milk is a complex system of different miRNA molecules with synergistic and antagonistic relationships, controlling specific immune responses in a lactating mammary gland. The stage of lactation and infection/inflammation has been shown to influence the miR-146a mediated epigenetic regulation of the immune response.

Thus, milk miR-146a can be used for the early detection of mastitis, which is in the reversible stage of the disease. Additionally, based on various studies, miR-146a, through different signalling pathways, can affect the expression system of related genes and have a dual role in cancer metastasis and progression.

### miR-383

miR-383 expression was increased in the milk of buffaloes with mastitis. Clinical mastitis had a greater expression level than subclinical mastitis. The data analysis demonstrated that the elevation of miR-383 differed substantially (*p* < 0.05) between buffaloes with subclinical and clinical mastitis and control/healthy buffaloes’ milk.

These results agree with Lai et al. (2017b) [8], who reported significant upregulation in miR-383 in CMT + ve milk of cows, which is known to be related to inflammation. miR-383 exhibited high predictive value. The dysregulation of miR-383 expression plays a role in inflammatory diseases and thus can be seen altered in mammary epithelial cells of mastitis-affected buffalo/cows. Similar to miR-146a, miR-383 in milk can also be a suitable candidate liquid biopsy biomarker for mastitis. For the first time, milk miR-383 was studied, and its relationship and involvement in the inflammatory response or inflammation in mastitis and upregulated expression were observed [37]. Our study validates their result with an average 11 to 25 fold change in subclinical and clinical mastitis milk, respectively. They reported that miR-383 dysregulation impacts cell activity in tumor cells. The upregulation of miR-383 indicates that it regulates the immune response by IL-1, TNF, COX-2, TLR4, and CXCL-1. The inflammation of the mammary gland in mastitis is a tissue reaction to infection caused by bacteria [46]. The evidence has shown a causal association between inflammation and cancer, and the inflammatory condition often precedes malignant change or accompanies a primary tumor of the mammary gland if mastitis is not treated in the early stages.

The adaptive changes in miR-383 in milk/mammary tissues in response to mastitis can elucidate how miRNAs modulate immune responses and provide appropriate strategies to control mastitis [47]. miR-383 promotes tumor cell apoptosis, while IL-17 reverses the positive effect of miR-383 on tumor cell apoptosis in carcinoma [48]. This may be similar in the process of mastitis, and upregulation of miR-383 in milk is an indicator for the process of mammary tumor, which can be detected at very early stages as it increases 2-fold even in normal milk having SCC ranging between 1 and 2 lakhs.

Similar to miR-146a, the upregulated expression of miR-383 may provide a potent target for the very early diagnosis of mastitis. It can be a meaningful reference for determining the clinical pathological stage to accept pertinent treatment promptly.

LINC00096 directly interacts with miR-383 and promotes proliferation and invasion by regulating the miR-383-5p/RBM3 pathway in breast cancer in humans [49]. This may be the case even in buffaloes with mastitis if not treated timely. The expression of miR-383 and miR-146a plays a vital role in inflammatory infections such as mastitis. The miRNA expression levels in cows with mastitis are altered in mammary epithelial cells, milk exosomes, and mammary gland tissue [14]. Both miR-383 and miR-146a have more gene targets related to inflammatory pathways than miRNA candidates. miR-383 mediates cell proliferation by regulating the expression of proliferation-associated genes [18]. Overexpression of miR-383 also induces cell cycle arrest in the G0/G1 phase and inhibits the proliferation of HepG2 and SK-Hep-1 cells. Moreover, miR-383 involves multiple signaling pathways. However, the apoptosis-promoting function of miR-383 is controversial.

### Correlation of somatic cell count with miR-146a and miR-383 expression

This is the first study wherein SCC has been correlated with miRNAs. Milk somatic cell count is a sensitive indicator of mammary gland inflammation. The relationship between SCC and miR-146a and miR-383 expression in the milk of normal buffalo milk was positively correlated. A similar positive correlation was also observed between SCC and mR-146a and miR-383 expression in buffaloes’ subclinical mastitis milk. This may be due to the start of an inflammatory reaction, indicating that miR-146a and miR-383 can be potential biomarkers for detecting mastitis at a very early stage.

The SCC, or characteristics derived from it, is frequently used to identify infected and uninfected mammary glands, as well as to monitor udder health [50]. The recruited leukocytes play an essential role in the defense of the mammary gland, as shown by the severe mastitis that develops when the leukocyte influx into milk is delayed or blocked. Neutrophils are necessary to protect against infections, which leads to a rise in somatic cell count, but in normal animals, the somatic cell count is so low that it will not be sufficient to trigger inflammation [51]. Mammary epithelial cells and their associated cells can detect the presence of bacteria and bacterial toxins [52–54]. They express many pattern recognition receptors that recognize bacterial ligands known as microbe-associated molecular patterns [55]. This is true at least for TLR2, TLR4, TLR9, and even the nucleotide-binding oligomerization domain (NOD)-like receptors NOD1 and NOD2 [56, 57]. MECs react to the microbe-associated molecular patterns they sense by producing several self-defense reagents (antimicrobial peptides and reactive oxygen species) and mediators of inflammation (cytokines and chemokines) [52, 53, 58, 59].

Thus, we conclude that miRNA quantification could enhance the accuracy of mastitis diagnosis, especially in cases where CMT might not provide a clear indication of subclinical mastitis. These miRNAs, miR-146a, and miR-383, could help identify inflammation and serve as valuable prognostic biomarkers for early subclinical mastitis detection, even in CMT-ve milk samples, indicating a potential avenue for improving mastitis detection methods. The effectiveness of using miRNA quantification for mastitis diagnosis would likely depend on further research and validation.

### Supplementary Information

**Additional file 1.** Concentration and Ratio of miRNA isolated from samples.

**Additional file 2.** cDNA Concentration/Ratio of miR-92a, miR-383 and miR-146a.

**Additional file 3.** Mean ± SE of SCC (x105 cells/ml) in normal/healthy, sub-clinical and clinical mastitis milk of buffaloes.

**Additional file 4.** Average Ct values, ΔCt values of sub-clinical mastitis group and control group, ΔΔCt and expression fold change in miR-146a.

**Additional file 5.** Average Ct values, ΔCt values of Clinical Mastitis group and control group, ΔΔCt and expression fold change in miR-146a.

**Additional file 6.** Independent T test for miR-146a between normal and sub-clinical mastitis.

**Additional file 7.** Independent T test for miR-146a between normal and Clinical mastitis.

**Additional file 8.** Average Ct values, ΔCt values of Sub-clinical Mastitis group and control group, ΔΔCt and expression fold change in miR-383.

**Additional file 9.** Average Ct values, ΔCt values of Clinical Mastitis group and control group, ΔΔCt and expression fold change in miR-383.

**Additional file 10.** Independent T test for miR-383 between normal and sub-clinical mastitis.

**Additional file 11.** Independent T test for miR-383 between normal and clinical mastitis.

**Additional file 12.** Intercorrelation matrix of SCC, miR-146a and miR-383 in normal milk of buffaloes.

**Additional file 13.** Intercorrelation matrix of SCC, miR-146a and miR-383 in sub-clinical mastitis milk of buffaloes.

**Additional file 14.** Intercorrelation matrix of SCC, miR-146a and miR-383 in clinical mastitis milk of buffaloes.

## Data Availability

The data supporting the results are available in the supplementary files.

## Abbreviations

SCC: Somatic Cell Count
mRNA: Messenger RNA
miRNA: MicroRNA
CMT: California Mastitis Test
ROC Curve: Receiver Operating Characteristic Curve
HIF1a: Hypoxia Inducible Factor 1-Alpha
VEGF: Vascular Endothelial Growth Factor
EMT: Epithelial Mesenchymal Transition
