# Supplementary material for: Milk miRNA expression in buffaloes as a potential biomarker for mastitis
Source: BMC Vet Res. 2024 Apr 20;20:150. doi: 10.1186/s12917-024-04002-1 (PMC11031985; doi:10.1186/s12917-024-04002-1)
Supplement: Supplementary file 1 — Additional file 1. Concentration and Ratio of miRNA isolated from samples. [file 12917_2024_4002_MOESM1_ESM.docx]

**Additional File 1:** Concentration and Ratio of miRNA isolated from samples.

| Buffalo/Sample no. | Group | Concentration | Ratio |
| --- | --- | --- | --- |
| 1 | Normal | 5.55µg/ml | 1.95 |
| 2 | Normal | 3.05µg/ml | 1.77 |
| 3 | Normal | 4.76µg/ml | 1.31 |
| 4 | Normal | 2.51µg/ml | 1.98 |
| 5 | Normal | 8.23µg/ml | 1.56 |
| 6 | Normal | 5.78µg/ml | 1.88 |
| 7 | Normal | 7.43µg/ml | 1.67 |
| 8 | Normal | 10.87µg/ml | 1.83 |
| 9 | Normal | 5.98µg/ml | 1.88 |
| 10 | Normal | 12.4µg/ml | 1.77 |
| 11 | Sub-clinical Mastitis | 6.568µg/ml | 1.55 |
| 12 | Sub-clinical Mastitis | 8.38µg/ml | 1.85 |
| 13 | Sub-clinical Mastitis | 4.29µg/ml | 1.37 |
| 14 | Sub-clinical Mastitis | 12.69µg/ml | 2.4 |
| 15 | Sub-clinical Mastitis | 7.18µg/ml | 3.5 |
| 16 | Sub-clinical Mastitis | 38.05µg/ml | 1.74 |
| 17 | Sub-clinical Mastitis | 8.08µg/ml | 1.72 |
| 18 | Sub-clinical Mastitis | 12.32 µg/ml | 1.87 |
| 19 | Sub-clinical Mastitis | 13.66µg/ml | 1.88 |
| 20 | Sub-clinical Mastitis | 29.05µg/ml | 2.11 |
| 21 | Clinical Mastitis | 42.68 µg/ml | 1.87 |
| 22 | Clinical Mastitis | 32.23 µg/ml | 1.36 |
| 23 | Clinical Mastitis | 7.2 µg/ml | 2 |
| 24 | Clinical Mastitis | 10.43 µg/ml | 1.88 |
| 25 | Clinical Mastitis | 6.86 µg/ml | 1.58 |
| 26 | Clinical Mastitis | 15.67 µg/ml | 1.45 |
| 27 | Clinical Mastitis | 23.67 µg/ml | 1.68 |
| 28 | Clinical Mastitis | 29.78 µg/ml | 1.89 |
| 29 | Clinical Mastitis | 25.89 µg/ml | 1.64 |
| 30 | Clinical Mastitis | 18.34 µg/ml | 1.95 |
