# Supplementary material for: Milk miRNA expression in buffaloes as a potential biomarker for mastitis
Source: BMC Vet Res. 2024 Apr 20;20:150. doi: 10.1186/s12917-024-04002-1 (PMC11031985; doi:10.1186/s12917-024-04002-1)
Supplement: Supplementary file 2 — Additional file 2. cDNA Concentration/Ratio of miR-92a, miR-383 and miR-146a. [file 12917_2024_4002_MOESM2_ESM.docx]

**Additional File 2:** cDNA Concentration/Ratio of miR-92a, miR-383 and miR-146a

| Buffalo/ Sample  No. | miR-92a  (Concentration/Ratio) | miR-383  (Concentration/Ratio) | miR-146a  (Concentration/Ratio) |
| --- | --- | --- | --- |
| 1 | 1355.7ng/µL / 1.81 | 1530.3ng/µL /1.83 | 2583.3ng/µL / 1.80 |
| 2 | 910ng/µL /1.81 | 1264.8ng/µL /1.83 | 834.1ng/µL /1.81 |
| 3 | 1264.5ng/µL /1.83 | 1133.7ng/µL /1.83 | 813.6ng/µL /1.81 |
| 4 | 1154.7ng/µL /1.82 | 1233ng/µL /1.83 | 938.3ng/µL /1.82 |
| 5 | 1726.1ng/µL /1.82 | 1167.4ng/µL /1.82 | 931ng/µL /1.80 |
| 6 | 1884.7ng/µL /1.83 | 1051ng/µL /1.833 | 1551.19ng/µL /1.835 |
| 7 | 1122.18ng/µL /1.831 | 1290.9ng/µL /1.834 | 1020.8ng/µL /1.828 |
| 8 | 1163.93ng/µL /1.82 | 1301.6ng/µL /1.833 | 1803.3ng/µL /1.84 |
| 9 | 1318 ng/µL/1.82 | 1328 ng/µL/1.832 | 1129 ng/µL/1.84 |
| 10 | 1352 ng/µL/1.83 | 1275 ng/µL/1.834 | 1185 ng/µL/1.82 |
| 11 | 1184 ng/µL/1.82 | 1466 ng/µL/1.823 | 2255 ng/µL/1.84 |
| 12 | 2000 ng/µL/1.82 | 1535 ng/µL/1.824 | 1585 ng/µL/1.86 |
| 13 | 1864 ng/µL/1.84 | 1629 ng/µL/1.825 | 1426 ng/µL/1.87 |
| 14 | 1765 ng/µL/1.82 | 1638 ng/µL/1.821 | 1317 ng/µL/1.88 |
| 15 | 1711 ng/µL/1.83 | 1459 ng/µL/1.826 | 1355 ng/µL/1.81 |
| 16 | 2211 ng/µL/1.83 | 2345 ng/µL/1.83 | 2106 ng/µL/1.86 |
| 17 | 1345 ng/µL/1.82 | 1567 ng/µL/1.87 | 1789 ng/µL/1.83 |
| 18 | 1100 ng/µL/1.80 | 1231 ng/µL/1.83 | 1178 ng/µL/1.88 |
| 19 | 890 ng/µL/1.81 | 821 ng/µL/1.84 | 700 ng/µL/1.81 |
| 20 | 768 ng/µL/1.82 | 890 ng/µL/1.86 | 876 ng/µL/1.83 |
| 21 | 1234 ng/µL/1.81 | 1567 ng/µL/1.84 | 1789 ng/µL/1.87 |
| 22 | 1342 ng/µL/1.82 | 1456 ng/µL/1.823 | 2007 ng/µL/1.89 |
| 23 | 1890 ng/µL/1.83 | 1345 ng/µL/1.86 | 2134 ng/µL/1.82 |
| 24 | 1765 ng/µL/1.82 | 1233 ng/µL/1.856 | 2090 ng/µL/1.87 |
| 25 | 1457 ng/µL/1.81 | 1687 ng/µL/1.86 | 1944 ng/µL/1.83 |
| 26 | 1888 ng/µL/1.82 | 1775 ng/µL/1.823 | 1389 ng/µL/1.82 |
| 27 | 1976 ng/µL/1.82 | 1453 ng/µL/1.833 | 2134 ng/µL/1.82 |
| 28 | 2167 ng/µL/1.83 | 2067 ng/µL/1.834 | 2000 ng/µL/1.85 |
| 29 | 1677 ng/µL/1.83 | 1389 ng/µL/1.87 | 1722 ng/µL/1.86 |
| 30 | 1100 ng/µL/1.82 | 989 ng/µL/1.89 | 936 ng/µL/1.821 |
