# Supplementary material for: Milk miRNA expression in buffaloes as a potential biomarker for mastitis
Source: BMC Vet Res. 2024 Apr 20;20:150. doi: 10.1186/s12917-024-04002-1 (PMC11031985; doi:10.1186/s12917-024-04002-1)
Supplement: Supplementary file 3 — Additional file 3. Mean ± SE of SCC (x105 cells/ml) in normal/healthy, sub-clinical and clinical mastitis milk of buffaloes. [file 12917_2024_4002_MOESM3_ESM.docx]

**Additional File 3: Mean ± SE of SCC (x10^5^ cells/ml) in normal/healthy, sub-clinical and clinical mastitis milk of buffaloes**.

| **Buffalo/Sample No.** | **Normal** | **Sub-clinical** | **Clinical** |
| --- | --- | --- | --- |
| 1, 11, 21 | 0.85 | 18.48 | 42.78 |
| 2, 12, 22 | 0.63 | 19.06 | 27.13 |
| 3. 13,23 | 0.73 | 7.60 | 45.09 |
| 4. 14, 24 | 0.88 | 8.06 | 24.03 |
| 5. 15. 25 | 0.96 | 17.84 | 30.19 |
| 6, 16, 26 | 0.95 | 11.79 | 47.03 |
| 7, 17. 27 | 1.10 | 4.25 | 48.73 |
| 8, 18, 28 | 1.45 | 8.46 | 29.46 |
| 9, 19, 29 | 1.23 | 6.07 | 41.02 |
| 10, 20. 30 | 0.56 | 4.87 | 37.8 |
| **Mean ± SE** | **0.93 ± 0.08 x 10^5^** | **10.64 ± 1.82x 10^5^** | **37.25 ± 2.8 x 10^5^** |
