# Supplementary material for: Milk miRNA expression in buffaloes as a potential biomarker for mastitis
Source: BMC Vet Res. 2024 Apr 20;20:150. doi: 10.1186/s12917-024-04002-1 (PMC11031985; doi:10.1186/s12917-024-04002-1)
Supplement: Supplementary file 4 — Additional file 4. Average Ct values, ΔCt values of sub-clinical mastitis group and control group, ΔΔCt and expression fold change in miR-146a. [file 12917_2024_4002_MOESM4_ESM.docx]

**Additional File 4: Average Ct values, ΔCt values of sub-clinical mastitis group and control group, ΔΔCt and expression fold change in miR-146a.**

| **Sample**  **name/no** | **Sub-clinical mastitis**  **CT values** | | **Control**  **CT values** | | **ΔC_t_ (Test)** | **ΔC_t_ (Control)** | **ΔΔC_t_**  **(test)** | **Fold change** |
| --- | --- | --- | --- | --- | --- | --- | --- | --- |
|  | miR-146a | miR-92a | miR-146a | miR-92a |  |  |  |  |
| 11 | 29.09 | 29.40 | 37.42 | 33.12 | -0.31 | 4.30 | -3.80 | 13.96 |
| 12 | 28.13 | 29.31 | 38.18 | 29.64 | -1.18 | 8.54 | -4.67 | 25.51 |
| 13 | 30.56 | 30.70 | 31.96 | 30.59 | -0.14 | 1.37 | -3.63 | 12.41 |
| 14 | 29.4 | 30.90 | 30.04 | 28.29 | -1.50 | 1.75 | -4.99 | 31.85 |
| 15 | 30.16 | 31.55 | 40.6 | 32.67 | -1.39 | 7.93 | -4.88 | 29.51 |
| 16 | 28.39 | 29.75 | 35 | 33.72 | -1.36 | 1.28 | -4.85 | 28.90 |
| 17 | 30.2 | 30.61 | 35.54 | 33.43 | -0.41 | 2.11 | -3.90 | 14.96 |
| 18 | 30.07 | 30.45 | 33.54 | 31.29 | -0.38 | 2.25 | -3.87 | 14.65 |
| 19 | 30.98 | 31.27 | 33.32 | 30.99 | -0.29 | 2.33 | -3.78 | 13.77 |
| 20 | 30.19 | 31.59 | 34.52 | 31.45 | -1.40 | 3.07 | -4.89 | 29.71 |
| **AVG** | **29.17** | **30.55** | **35.1** | **31.53** | **-0.836** | **3.49** | **-0.836** | **21.52** |
