# Supplementary material for: Milk miRNA expression in buffaloes as a potential biomarker for mastitis
Source: BMC Vet Res. 2024 Apr 20;20:150. doi: 10.1186/s12917-024-04002-1 (PMC11031985; doi:10.1186/s12917-024-04002-1)
Supplement: Supplementary file 5 — Additional file 5. Average Ct values, ΔCt values of Clinical Mastitis group and control group, ΔΔCt and expression fold change in miR-146a. [file 12917_2024_4002_MOESM5_ESM.docx]

**Additional File 5: Average Ct values, ΔCt values of Clinical Mastitis group and control**

**group, ΔΔCt and expression fold change in miR-146a.**

| **Sample**  **name/no** | **Clinical mastitis**  **CT values** | | **Control**  **CT values** | | **ΔC_t_ (Test)** | **ΔC_t_ (Control)** | **ΔΔC_t_**  **(test)** | **Fold change** |
| --- | --- | --- | --- | --- | --- | --- | --- | --- |
|  | miR-146a | miR-92a | miR-146a | miR-92a |  |  |  |  |
| 21 | 26.22 | 29.23 | 37.42 | 33.12 | -3.01 | 4.30 | -6.50 | 90.70 |
| 22 | 30.04 | 33.15 | 38.18 | 29.64 | -3.11 | 8.54 | -6.60 | 97.21 |
| 23 | 26.79 | 30.04 | 31.96 | 30.59 | -3.25 | 1.37 | -6.74 | 107.11 |
| 24 | 27.64 | 31.37 | 30.04 | 28.29 | -3.73 | 1.75 | -7.22 | 149.40 |
| 25 | 26.8 | 30.22 | 40.6 | 32.67 | -3.42 | 7.93 | -6.91 | 120.51 |
| 26 | 27.96 | 31.23 | 35 | 33.72 | -3.27 | 1.28 | -6.76 | 108.61 |
| 27 | 28.74 | 32.15 | 35.54 | 33.43 | -3.41 | 2.11 | -6.90 | 119.68 |
| 28 | 27.51 | 30.87 | 33.54 | 31.29 | -3.36 | 2.25 | -6.85 | 115.60 |
| 29 | 27.14 | 29.62 | 33.32 | 30.99 | -2.48 | 2.33 | -5.97 | 62.81 |
| 30 | 26.92 | 29.87 | 34.52 | 31.45 | -2.95 | 3.07 | -6.44 | 87.00 |
| **AVG** | **25.57** | **30.7** | **35.01** | **31.53** | **-3.1** | **3.49** | **-6.69** | **105.86** |
