# Supplementary material for: Milk miRNA expression in buffaloes as a potential biomarker for mastitis
Source: BMC Vet Res. 2024 Apr 20;20:150. doi: 10.1186/s12917-024-04002-1 (PMC11031985; doi:10.1186/s12917-024-04002-1)
Supplement: Supplementary file 7 — Additional file 7. Independent T test for miR-146a between normal and Clinical mastitis. [file 12917_2024_4002_MOESM7_ESM.docx]

**Additional File 7: Independent T test for miR-146a between normal and Clinical mastitis.**

|  | | | | | |
| --- | --- | --- | --- | --- | --- |
|  | | Levene's Test for Equality of Variances | | t-test for Equality of Means | |
|  |  | F | Sig. | T | Df |
|  |  |  |  |  |  |
| value | Equal variances assumed | 109.083 | .000 | -7.359 | 18 |
|  | Equal variances not assumed |  |  | -7.359 | 9.746 |
