# Supplementary material for: Milk miRNA expression in buffaloes as a potential biomarker for mastitis
Source: BMC Vet Res. 2024 Apr 20;20:150. doi: 10.1186/s12917-024-04002-1 (PMC11031985; doi:10.1186/s12917-024-04002-1)
Supplement: Supplementary file 8 — Additional file 8. Average Ct values, ΔCt values of Sub-clinical Mastitis group and control group, ΔΔCt and expression fold change in miR-383. [file 12917_2024_4002_MOESM8_ESM.docx]

**Additional File 8: Average Ct values, ΔCt values of Sub-clinical Mastitis group and control group, ΔΔCt and expression fold change in miR-383.**

| **Sample**  **name/no** | **Sub-Clinical mastitis**  **CT values** | | **Control**  **CT values** | | **ΔC_t_ (Test)** | **ΔC_t_ (Control)** | **ΔΔC_t_**  **(test)** | **Fold change** |
| --- | --- | --- | --- | --- | --- | --- | --- | --- |
|  | miR-383 | miR-92a | miR-383 | miR-92a |  |  |  |  |
| 11 | 29.14 | 27.40 | 36.92 | 33.12 | 1.74 | 3.80 | -3.64 | 12.47 |
| 12 | 29.5 | 28.31 | 35.72 | 29.64 | 1.19 | 6.08 | -4.19 | 18.25 |
| 13 | 33.28 | 31.70 | 36.12 | 30.59 | 1.58 | 5.53 | -3.80 | 13.93 |
| 14 | 34.1 | 30.90 | 33.82 | 28.29 | 3.20 | 5.53 | -2.18 | 4.53 |
| 15 | 30.2 | 28.55 | 36.6 | 32.67 | 1.65 | 3.93 | -3.73 | 13.27 |
| 16 | 31.02 | 29.75 | 36.78 | 31.72 | 1.27 | 5.06 | -4.11 | 17.27 |
| 17 | 33.31 | 30.61 | 36.74 | 33.43 | 2.70 | 3.31 | -2.68 | 6.41 |
| 18 | 33.72 | 30.45 | 38.32 | 31.29 | 3.27 | 7.03 | -2.11 | 4.32 |
| 19 | 33.13 | 31.27 | 38.45 | 30.99 | 1.86 | 7.46 | -3.52 | 11.47 |
| 20 | 30.41 | 30.59 | 37.52 | 31.45 | 1.82 | 6.07 | -3.56 | 11.79 |
| **AVG** | **31.98** | **29.95** | **36.69** | **31.32** | **2.03** | **5.38** | **-3.35** | **11.37** |
