# Supplementary material for: Milk miRNA expression in buffaloes as a potential biomarker for mastitis
Source: BMC Vet Res. 2024 Apr 20;20:150. doi: 10.1186/s12917-024-04002-1 (PMC11031985; doi:10.1186/s12917-024-04002-1)
Supplement: Supplementary file 9 — Additional file 9. Average Ct values, ΔCt values of Clinical Mastitis group and control group, ΔΔCt and expression fold change in miR-383. [file 12917_2024_4002_MOESM9_ESM.docx]

**Additional File 9: Average Ct values, ΔCt values of Clinical Mastitis group and control group, ΔΔCt and expression fold change in miR-383.**

| **Sample no/name** | **Clinical mastitis**  **CT values** | | **Control**  **CT values** | | **ΔC_t_ (Test)** | **ΔC_t_ (Control)** | **ΔΔC_t_**  **(test)** | **Fold change** |
| --- | --- | --- | --- | --- | --- | --- | --- | --- |
|  | miR-383 | miR-92a | miR-383 | miR-92a |  |  |  |  |
| 21 | 33.34 | 31.98 | 36.92 | 33.12 | 1.36 | 3.80 | -4.02 | 16.22 |
| 22 | 31.05 | 31.78 | 35.72 | 29.64 | -0.73 | 6.08 | -6.11 | 69.07 |
| 23 | 27.68 | 28.09 | 36.12 | 30.59 | -0.41 | 5.53 | -5.79 | 55.33 |
| 24 | 31.38 | 29.37 | 33.82 | 28.29 | 2.01 | 5.53 | -3.37 | 10.34 |
| 25 | 28.5 | 27.22 | 36.6 | 32.67 | 1.28 | 3.93 | -4.10 | 17.15 |
| 26 | 32.02 | 30.23 | 36.78 | 31.72 | 1.79 | 5.06 | -3.59 | 12.04 |
| 27 | 30.54 | 29.10 | 36.74 | 33.43 | 1.44 | 3.31 | -3.94 | 15.35 |
| 28 | 30.1 | 28.32 | 38.32 | 31.29 | 1.78 | 7.03 | -3.60 | 12.13 |
| 29 | 27.12 | 26.62 | 38.45 | 30.99 | 0.50 | 7.46 | -4.88 | 29.45 |
| 30 | 28.64 | 26.92 | 37.52 | 31.45 | 1.72 | 6.07 | -3.66 | 12.64 |
| **AVG** | **30.03** | **28.96** | **36.69** | **31.32** | **1.07** | **5.38** | **-4.31** | **24.97** |
