# Supplementary material for: Milk miRNA expression in buffaloes as a potential biomarker for mastitis
Source: BMC Vet Res. 2024 Apr 20;20:150. doi: 10.1186/s12917-024-04002-1 (PMC11031985; doi:10.1186/s12917-024-04002-1)
Supplement: Supplementary file 10 — Additional file 10. Independent T test for miR-383 between normal and sub-clinical mastitis. [file 12917_2024_4002_MOESM10_ESM.docx]

**Additional File 10: Independent T test for miR-383 between normal and sub-clinical mastitis**

|  | | | | | |
| --- | --- | --- | --- | --- | --- |
|  | | Levene's Test for Equality of Variances | | t-test for Equality of Means | |
|  |  | F | Sig. | T | Df |
|  |  |  |  |  |  |
| Value | Equal variances assumed | 8.361 | .010 | -6.174 | 18 |
|  | Equal variances not assumed |  |  | -6.174 | 10.361 |
