# Supplementary material for: Milk miRNA expression in buffaloes as a potential biomarker for mastitis
Source: BMC Vet Res. 2024 Apr 20;20:150. doi: 10.1186/s12917-024-04002-1 (PMC11031985; doi:10.1186/s12917-024-04002-1)
Supplement: Supplementary file 12 — Additional file 12. Intercorrelation matrix of SCC, miR-146a and miR-383 in normal milk of buffaloes. [file 12917_2024_4002_MOESM12_ESM.docx]

| **Additional File 12: Intercorrelation matrix of SCC, miR-146a and miR-383 in normal milk of buffaloes**. | | | | |
| --- | --- | --- | --- | --- |
|  | | SCC | miR-146a | miR-383 |
| SCC | Pearson Correlation | 1 | .197 | .258 |
|  | Sig. (2-tailed) |  | .586 | .472 |
|  | N | 10 | 10 | 10 |
| G1 | Pearson Correlation | .197 | 1 | -.206 |
|  | Sig. (2-tailed) | .586 |  | .567 |
|  | N | 10 | 10 | 10 |
| G2 | Pearson Correlation | .258 | -.206 | 1 |
|  | Sig. (2-tailed) | .472 | .567 |  |
|  | N | 10 | 10 | 10 |
