# Supplementary material for: Milk miRNA expression in buffaloes as a potential biomarker for mastitis
Source: BMC Vet Res. 2024 Apr 20;20:150. doi: 10.1186/s12917-024-04002-1 (PMC11031985; doi:10.1186/s12917-024-04002-1)
Supplement: Supplementary file 14 — Additional file 14. Intercorrelation matrix of SCC, miR-146a and miR-383 in clinical mastitis milk of buffaloes. [file 12917_2024_4002_MOESM14_ESM.docx]

**Additional File 14: Intercorrelation matrix of SCC, miR-146a and miR-383 in clinical mastitis milk of buffaloes.**

|  | | SCC | miR-146a | miR-383 |
| --- | --- | --- | --- | --- |
| SCC | Pearson Correlation | 1 | -.378 | -.078 |
|  | Sig. (2-tailed) |  | .282 | .831 |
|  | N | 10 | 10 | 10 |
| G1 | Pearson Correlation | -.378 | 1 | -.281 |
|  | Sig. (2-tailed) | .282 |  | .432 |
|  | N | 10 | 10 | 10 |
| G2 | Pearson Correlation | -.078 | -.281 | 1 |
|  | Sig. (2-tailed) | .831 | .432 |  |
|  | N | 10 | 10 | 10 |
